# Geriatrician perspectives on perioperative care: a qualitative study

**DOI:** 10.1186/s12877-021-02019-x

**Published:** 2021-01-19

**Authors:** Janani Thillainadesan, Jesse Jansen, Jacqui Close, Sarah Hilmer, Vasi Naganathan

**Affiliations:** 1 Department of Geriatric Medicine, Concord Hospital, Sydney, Australia; 2 Concord Clinical School, Faculty of Medicine and Health, University of Sydney, Sydney, Australia; 3 Centre for Education and Research on Ageing, and Ageing and Alzheimers Institute, Sydney, Australia; 4 Centre Wiser Healthcare, and Sydney Health Literacy Lab, School of Public Health, Faculty of Medicine and Health, University of Sydney, Sydney, Australia; 5 Falls, Balance and Injury Research Centre, Neuroscience Research Australia, University of New South Wales, and Prince of Wales Clinical School, University of New South Wales, Sydney, Australia; 6 Kolling Institute of Medical Research, Sydney Medical School, University of Sydney and Royal North Shore Hospital, Sydney, Australia

**Keywords:** Health services for the aged, Perioperative care, Qualitative research, Geriatricians

## Abstract

**Background:**

Perioperative medicine services for older surgical patients are being developed across several countries. This qualitative study aims to explore geriatricians’ perspectives on challenges and opportunities for developing and delivering integrated geriatrics perioperative medicine services.

**Methods:**

A qualitative phenomenological semi-structured interview design. All geriatric medicine departments in acute public hospitals across Australia and New Zealand (*n* = 81) were approached. Interviews were conducted with 38 geriatricians. Data were analysed thematically using a framework approach.

**Results:**

Geriatricians identified several system level barriers to developing geriatrics perioperative medicine services. These included lack of funding for staffing, encroaching on existing consultative services, and competing clinical priorities. The key barrier at the healthcare professional level was the current lack of clarity of roles within the perioperative care team. Key facilitators were perceived unmet patient needs, existing support for geriatrician involvement from surgical and anaesthetic colleagues, and the unique skills geriatricians can bring to perioperative care. Despite reporting barriers, geriatricians are contemplating and implementing integrated proactive perioperative medicine services. Geriatricians identified a need to support other specialties gain clinical experience in geriatric medicine and called for pragmatic research to inform service development.

**Conclusions:**

Geriatricians perceive several challenges at the system and healthcare professional levels that are impacting current development of geriatrics perioperative medicine services. Yet their strong belief that patient needs can be met with their specialty skills and their high regard for team-based care, has created opportunities to implement innovative multidisciplinary models of care for older surgical patients. The barriers and evidence gaps highlighted in this study may be addressed by qualitative and implementation science research. Future work in this area may include application of patient-reported measures and qualitative research with patients to inform patient-centred perioperative care.

**Supplementary Information:**

The online version contains supplementary material available at 10.1186/s12877-021-02019-x.

## Background

Due to the ageing population and the advent of new less-invasive surgical and anaesthetic techniques, more older adults are undergoing surgical procedures [1, 2]. Therefore surgeons are looking after older and more complex patients who have multimorbidity, polypharmacy, frailty and other geriatric syndromes which increase their risk of perioperative morbidity and mortality [3–5]. This has seen the emergence of perioperative medicine for older patients [6], where a multidisciplinary surgical team assesses, manages and optimises the patient throughout the continuum of care from the decision to operate to long-term recovery. While traditional perioperative management has involved optimisation of single-organ systems or specific diseases (e.g. diabetes), the growing recognition of the complex care needs of older adults undergoing surgery has seen recent international perioperative care guidelines recommend proactive management of geriatric syndromes such as delirium, frailty and functional impairment [7, 8]. Accordingly, new perioperative interventions and models of care are being developed that embed multidisciplinary teams and target geriatric syndromes [9, 10].

A long-standing example of a multidisciplinary integrated model of care for older surgical patients is orthogeriatrics care. Developed by orthopaedic surgeon Dr. Devas in the 1970s, orthogeriatrics care is now well-established and proven [10–12]. However, in many hospitals older non-orthopaedic surgical patients receive geriatrician input through a traditional consultation model of care [13–15]. This model typically involves a nurse-led preoperative assessment, sometimes followed by an anaesthetics review. Referrals to other specialties occur as required and often ‘reactively’, that is, after medical complications including geriatrics syndromes have developed. Evidence for geriatrics surgical collaborative models of care beyond orthogeriatrics is emerging [9, 10, 16]. In these models of care a dedicated geriatric medicine team proactively undertakes comprehensive geriatric assessment and management (CGA) on all patients whom fulfil a pre-determined criteria such as older age, multiple comorbidities or frailty.

In a recent survey of 67 geriatricians across Australia and New Zealand, all participants believed there was a need for geriatricians to provide greater input into the care of older surgical patients [15]. In the UK there has been an increase in the number of geriatric medicine services for older surgical patients from 38 (29.2%) in 2013 to 81 (53.3%) in 2018 [13, 14]. With recent national and international emphasis on collaborative perioperative models of care [7, 8], and emerging evidence for these models [9, 10, 16], this study was planned to explore the challenges and opportunities to develop and deliver geriatrics perioperative medicine services from the geriatrician’s perspective. Surveys of geriatricians have been undertaken to examine the state of perioperative medicine services using quantitative methods [13–15]. This study employed *qualitative* methods to explore in-depth the views and experiences of geriatricians that are not captured by quantitative methods and surveys [17].

## Methods

### Design

A qualitative phenomenological semi-structured interview design was used [18]. Ethical approval was granted by the Sydney Local Health District Human Research Ethics Committee at Concord Hospital (LNR/18/CRGH/90).

### Sampling and recruitment

All heads of geriatric medicine departments across Australia and New Zealand were approached via email. A list of hospitals was sourced from the Royal Australasian College of Physicians and was verified by geriatricians in each region. Eighty-one identified heads of departments were emailed a letter of invitation and asked to nominate themselves or a proxy, who was the geriatrician involved in providing perioperative medicine services in their department. Written consent was provided via a secure REDCap web link and consent was reconfirmed verbally at the start of the interview for all participants. Telephone interviews were scheduled with consenting geriatricians and were conducted between June 2019 and September 2019.

### Data collection

The study team consisted of geriatricians and academics with qualitative research experience. To achieve consistency of questioning, one investigator (JT) undertook all interviews. The interview guide was developed for this study, and was based on findings from a recent survey of geriatricians about perioperative medicine services [15] and the literature (see supplementary File 1). The guide included questions about service provision and training needs. The interview guide was pilot-tested to ensure clarity, timing and wording of questions. Interviews were continued until the sample size was sufficiently large and varied to explore the aims of the study [19]. Field notes were taken during the interview and notable comments were recorded verbatim.

### Data analysis

A framework analysis was conducted to identify themes that captured the range of views expressed [20]. Two investigators (JT and VN) independently reviewed and coded each interview transcript using inductive and deductive coding. Investigators met to refine codes, identify themes, and develop a working analytical framework. An audit trail was produced by maintaining a record of coding decisions [21]. After analysis of all transcripts, data were charted into a final framework matrix. The matrix was serially reviewed and interpreted both independently by JT and as a team. In the final refined analysis, subthemes were combined under thematic headings. Data were managed and analysed using Microsoft Excel.

## Results

Thirty-eight geriatricians were interviewed. The median (interquartile range) number of years working as a geriatrician was 18 (6–27) years. Twenty-nine were from Australia (76.3%), and nine (23.7%) from New Zealand. Thirty-one (84%) geriatricians consulted on surgical patients as part of their current clinical duties.

Analysis of interviews revealed three key themes: (1) perceived barriers and facilitators to delivering perioperative care services, (2) models of care to deliver geriatrics perioperative medicine services and (3) education and research infrastructure. These thematic headings have direct implications for informing how geriatrics perioperative medicine services can be developed and delivered. The results are reported in the context of each thematic heading with representative quotations.

### Perceived barriers and facilitators to delivering perioperative care services

Geriatricians identified barriers and facilitators at the system, patient and healthcare professional levels.

### System level barriers and facilitators

Lack of funding for more geriatricians, geriatric medicine fellows and nurses emerged as the main barrier to becoming involved in perioperative medicine services. The current pay system is based on a fixed salary rather than a fee-for-service basis which means any expansion of service would increase workload without increased pay.

‘*They said it would just be a few more patients a week, but in fact my workload has now doubled but same pay.*’ *Participant 20*

‘*Clinic has no nursing support would be lucky to get a BP, ECG and weight.*’ *Participant 6*

Geriatricians provided examples of where they had been approached by surgeons or anaesthetists to get involved in surgical services but there was no funding available from existing geriatric medicine or surgical budgets. A few remarked on needing funds from other sources such as the general hospital budget or external grants.

*‘Neurosurgeons here are keen for a collaborative service and we put in an application, a business plan but unfortunately there was no additional funding in the system to support this.’ Participant 39*

A local system barrier cited by some was concern that the introduction of a proactive geriatrics perioperative medicine service or greater involvement of geriatricians in existing perioperative services would encroach on existing ‘reactive’ consultative services provided by general medicine.

*‘We have great potential, there are barriers with general medicine not wanting to let geriatric medicine expand.’ Participant 9*

*‘We already have a periop medical service here [delivered by general medicine] and I don’t want a turf war.’ Participant 60*

Some reported there were competing priorities between providing more perioperative care and delivering other aged care services such as community care and memory clinics.

*‘There are other areas like falls, balance and incontinence to also provide services in.’ Participant 8*

The key facilitator identified was good buy in from surgical and anaesthetic departments. A few geriatricians commented on working with “young” interested anaesthetists. This suggests changing attitudes over time and greater openness to collaborative care.

*‘So one of the other geriatricians here got in touch with an enthusiastic anaesthetist here who has an interest in older patients and also an interested surgeon and we now have a periop working group.’ Participant 38*

*‘They are interested in this. Being a new hospital we have a young dynamic group of anaesthetists, and orthogeris is embedded in their psyche.’ Participant 53*

One geriatrician reported their geriatrics perioperative medicine service started after they were approached by hospital management.

*‘We were first approached by my hospital management as they [colorectal surgery patients] have longest hospital stay in winter so as part of their winter strategy we thought we would start this service.’ Participant 16*

### Patient level facilitator

Geriatricians were primarily motivated by concerns about patient needs. They spoke about the older surgical patient’s complex care needs due to the presence of multimorbidity, cognitive impairment and frailty, while age itself was not mentioned.

*‘Elderly people have diminished reserve and frailty.’ Participant 3*

Some felt there were subgroups of surgical patients who were more vulnerable to being acutely unwell and having geriatric syndromes.

*‘Emergency are a different group, they are more sick, we need to look after all of them.’ Participant 16*

*‘They [vascular surgery patients] are often the most sick, they are diabetic, with chronic kidney disease, they are vascularpaths. They are sitting there with their necrotic toe but everything else is falling apart and they often have underlying undiagnosed cognitive impairment’. Participant 38*

While most perceptions of patient need were informed by personal and local experiences, some also referred to patient data.

*‘from the conference … data that showed when they looked at frailty the vascular patients are more frail’ Participant 35*

### Healthcare professional level barriers and facilitators

At a healthcare professional level, a key facilitator was the geriatricians’ strong belief that they had specialised skills to manage the complex acute and long-term care needs of older surgical patients. They talked about managing multimorbidity and complications, preventing, identifying and managing delirium, deconditioning and functional decline, as well as prognostication.

*‘Lots of old patients, chronic conditions that affect cognition, mobility, pain, they have multimorbidity and polypharmacy, and need geriatric medicine input.’ Participant 1*

*‘Geriatricians are best placed to follow the patient through the whole journey, we clearly have a role to play.’ Participant 49*

Some felt there were gaps in existing services such as meeting the care needs of frail patients and providing long-term follow-up, and this was a facilitator for geriatrician involvement.

*‘they [general medicine] use a medical model but not focussed on frail patients, they just manage them like acute problem.’ Participant 45*

*‘Periop medicine run by gen med here but they to do initial consult where they advise but not follow up.’ Participant 9*

The opportunity to collaborate with other specialists to deliver integrated patient care was perceived as an important facilitator. Geriatricians spoke of the unique and complementary skills of each specialist and how these could be applied to meet the different needs of patients across the continuum of care.

*‘I think the anaesthetist and geriatrician can bring their own skills mix and they can work together very well.’ Participant 36*

*‘Anaesthetists have a role to play, especially expertise immediately post-op and operation period, analgesia.’ Participant 8*

Geriatricians also felt a team approach to care offered opportunities for learning.

*‘We improve our knowledge of each other’s disciplines.’ Participant 6*

*‘Orthopaedic surgeons increasingly refer to us preoperatively, so they’re starting to pick frailty in a way.’ Participant 53*

At the same time, many geriatricians expressed concern about a lack of role delineation between the various specialties.

*‘Needs to be team environment with surgeons having defined role, anaesthetist and geris at parts of journey. Not to duplicate.’ Participant 8*

This lack of role delineation was the main barrier at a health professional level. Geriatricians felt they had unique skills that could not be assumed by other specialties. For example, geriatricians stated that anaesthetists were not best skilled to provide post-acute care because of the nature of the clinical care required during this period such as “to manage delirium”, “attending discharge meetings”, “prognostication” and “running family meetings”.

*‘I know anaesthetists are interested but I don’t think they are best placed, there’s an awful lot of functional stuff that happens post op, the physiological stuff is what anaesthetists are good at.’ Participant 15*

*‘I think it could be very useful to have anaesthetists train in caring for older people, but they cannot take on the role of a geriatrician as skills mix is very different.’ Participant 36*

One geriatrician spoke of their skills being undervalued in the perioperative setting.

*‘Our expertise in complexity with multimorbidity and patients declining, have not been recognised.’ Participant 6*

### Models of care to deliver geriatrics perioperative medicine services

When discussing what types of models of care could be implemented to deliver integrated geriatrics perioperative medicine services, a number of models were suggested which are summarised in Table 1. There were mixed opinions on which patient care setting (inpatient vs outpatient) geriatricians should start in given limited funding to expand geriatric medicine staffing.

**Table 1:** Geriatrics perioperative models of care that are currently being developed or implemented

**INPATIENT MODELS OF CARE**				
**Models of perioperative care**	**Setting**	**Referral process for geriatrician input**	**Leadership**	**Team members**
Traditional reactive model	Inpatient surgical ward	Review on request	Surgeon	Surgeon-led multidisciplinary nursing and allied health team +/− other specialist reviews as requested
Geriatrician on the ward	Inpatient surgical ward	Geriatrician present on the ward regularly e.g. weekly ward rounds	Surgeon	Surgeon-led multidisciplinary nursing and allied health team PLUS Geriatrician and/or an aged care trained allied health professional or nurse
Screening-based referral for Geriatrician review	Inpatient surgical ward +/− Emergency department	Non-geriatrician screens patients (e.g. frailty screening) and if specified criteria met referral made for Geriatrician review	Surgeon	Surgeon-led multidisciplinary nursing and allied health team PLUS Screening by nurse/allied health professional/anaesthetist +/− Geriatrician review if screening criteria met
Proactive case-finding by Geriatrician	Inpatient surgical ward +/− Emergency department	Geriatrician or aged care nurse attends ED, board meetings or case conferences to identify patients for Geriatrician review	Surgeon	Surgeon-led multidisciplinary nursing and allied health team +/− Geriatrician and/or aged care nurse review
Geriatrician Co-management	Inpatient surgical ward	Blanket referral and involvement from admission to discharge	Surgeon and Geriatrician	Surgeon and Geriatrician Co-management within a shared multidisciplinary team
**OUTPATIENT MODELS OF CARE**				
**Models of perioperative care**	**Setting**	**Referral process for geriatrician input**	**Leadership**	**Team members**
Traditional reactive model	Outpatient preadmission clinic	Non existent	Surgeon and/or Anaesthetist	Surgeon, anaesthetist and clinic nurse
Screening-based referral for Geriatrician review	Outpatient preadmission clinic +/− Inpatient surgical ward	Anaesthetist screens patients (e.g. frailty screening or presence of multimorbidity) and if specified criteria met referral made for Geriatrician review	Anaesthetist	Surgeon, anaesthetist and clinic nurse +/− Geriatrician and/or Aged Care nurse preoperative clinic review +/− Geriatrician and/or Aged Care nurse postoperative review on the ward
Joint preoperative clinic	Outpatient preoperative clinic +/− Inpatient surgical ward	Blanket referral	Geriatrician and Surgeon/Anaesthetist	Surgeon, geriatrician, anaesthetist and clinic nurse +/− Geriatrician postoperative review on the ward

### Proactive models of care

Some geriatricians were developing or had recently implemented proactive geriatrics perioperative services, while others had ideas for models (Table 1). The diverse range of models put forward mostly involved a multidisciplinary team. Inpatient models of care included services based on blanket referrals to geriatrics, screening-based referrals (e.g. frailty screening), or proactive case-finding of high-risk patients by geriatrics.

*‘We designed a service where they are screened for frailty in the emergency department.’ Participant 45*

*‘Could start as consultative for all > 80 years, and then triage into who may or may not need regular input after one review, or if complex have shared care.’ Participant 27*

The main outpatient service discussed was a geriatrician preadmission clinic. A few mentioned other initiatives including enhanced recovery after surgery and prehabilitation.

*‘We have a dedicated frailty nurse who triages from the preadmission clinic. If they score high on the Rockwood scale which anyone can do they are flagged for review by the frailty nurse who then reviews the patient, identifies any issues, and if they feel they need geriatrician input they refer to us.’ Participant 16*

*‘Joint anaesthetic geriatric pre-op clinic, mainly elective joint, elective colorectal. Referrals are triaged by the high-risk anaesthetist who is physician trained. [I] often see those with multimorbidity, functional problems.’ Participant 6*

### Starting a perioperative service in preadmission clinic versus inpatient setting

Geriatricians discussed the pros and cons of starting a service in preadmission clinic vs inpatient setting in the context of limited staffing. Reasons cited for starting with an inpatient service included: anticipated greater impact, geriatricians had specialist expertise to manage postoperative issues such as delirium, deconditioning and complications, and this would be similar to orthogeriatrics, an evidence-based model of care. Some felt that preoperative geriatrician clinics may encroach on the preadmission clinics run by anaesthetists, and there was often not enough time to optimise patients by the time they presented to the preoperative clinic.

*‘The current need is in inpatient care so the post op delirium, and post op medical problems and that’s where would start having discussions. On the other hand, could start with preadmissions clinic but it’s so vast and often by the time they get to preadmission the decision seems to be already made.’ Participant 38*

Those who favoured starting with a preoperative outpatient service thought this would allow early optimisation, ability to influence the decision to operate, build on pre-existing discussions with surgeons and anaesthetists to identify those for referral such as using frailty screening, and there were pre-existing successful preoperative models of care such as the Proactive Care of Older Patients Undergoing Surgery (POPS) service [16].

*‘I would like to do it properly like the POPS model, by being involved preoperatively and perioperatively and postoperatively in fast tracking and sending home … .but I don’t see it will be just perioperative medicine, it will be whole journey.’ Participant 49*

### Education and research infrastructure for perioperative medicine

Geriatricians’ views on education and training needs were explored. These needs are described at the registrar trainee level and consultant (post fellowship) level. Weak research infrastructure also emerged as an important issue.

### Perioperative medicine in the geriatric medicine training program

Geriatricians were asked whether the current geriatric medicine training program adequately prepared trainees for perioperative medicine consultation. In Australia and New Zealand, geriatric medicine is a subspecialty within internal medicine where the trainee completes 3 years of training in general, acute, and speciality care medicine followed by 3 years in geriatric medicine. While perioperative medicine is not a standard part of training, most trainees complete an orthogeriatrics, acute inpatient care and surgical consultation rotation. Most geriatricians felt that the current training program adequately prepared trainees for perioperative medicine consultation.

*‘As we do acute geriatrics, no major problem then to be able to manage postoperative delirium, medication management. And they also get orthogeriatrics training.’ Participant 27*

A few geriatricians expressed that orthogeriatrics training may not be enough, and some thought more training opportunities may be useful.

*‘Orthogeriatrics would not be enough as needs of general surgery, abdominal surgery are a different kettle of fish and post op fluid and electrolyte derangements is different, much more challenging than hip fractures, they might want realistically more ICU rotations than just orthogeris.’ Participant 48*

### Post fellowship perioperative medicine training qualification

Geriatricians were asked about the need to introduce a post fellowship perioperative medicine training qualification. Many appeared to be uncertain or indifferent while a few thought the additional qualification would be helpful for those interested and for early career geriatricians.

*‘There are those out there calling themselves periop physicians but no formal qualification. But then there are also excellent orthogeriatricians out there who have been working for years in the field, and I think experience counts a lot. But a fellowship would help.’ Participant 43*

*‘I think you would need to look at it carefully. As a speciality we’ve never really gone down the subspecialty path … I would approach it with caution.’ Participant 47*

Some geriatricians expressed a strong view about not introducing a perioperative medicine training qualification. The concerns raised were that this would lead to unnecessary overspecialisation, undermine the unique skills of geriatricians, and geriatricians already had the skills and knowledge to practice in perioperative medicine, and they were not interested in taking on other perioperative roles such as providing anaesthesia.

*‘It’s fundamentally what geriatricians do, so I would not think we need a qualification for that.’ Participant 35*

*‘This idea we all need to have the exact same skills is not right. I don’t want to look after people in theatre.’ Participant 6*

If a post fellowship perioperative medicine training qualification was introduced, geriatricians felt it should be accessible to all interested clinicians, and in particular anaesthetists, geriatricians and general physicians. Some also expressed that the qualification should teach geriatric medicine principles and offer geriatric medicine rotations for interested anaesthetists.

*‘I think if they want to look after older patients they need to do some geriatric medicine training too. A lot are not attuned to the needs of older patients. It’s a two way street. We are happy to do additional training.’ Participant 39*

### Research in perioperative medicine

One of the emerging themes was on the need for research in perioperative medicine. References were made to international initiatives including POPS and National Emergency Laparotomy Audit, and the well-established Australian and New Zealand hip fracture registry [16, 22].

*‘I think it’s really important to start collecting data when developing these services, for example we have recently joined the hip fracture registry.’ Participant 43*

Geriatricians based at smaller sites called for more real world clinical data to inform how to implement services using limited resources.

*‘There is evidence from some of the big centres where this research is done, but little evidence for smaller hospitals, and where resources are limited.’ Participant 35*

A few geriatricians believed that the evidence for proactive perioperative medicine services was not yet well established.

*‘Many people try to get funding before showing service works, but I think we need to try the service and show it works to get funding.’ Participant 10*

## Discussion

Momentum for integrated proactive perioperative services for older patients is growing across several countries [6]. A recent survey showed that geriatricians perceive a need to provide greater input into the care of older surgical patients [15]. There is also a growing evidence base for geriatrician embedded perioperative models of care. Yet there is still a significant gap in the implementation of these new models of care [15]. This study sheds light on the barriers and facilitators to implementing these models. Geriatricians’ perceptions of the needs of older surgical patients and the skills they can contribute to perioperative care are also highlighted. Insights are provided on potential models of care, research gaps and training needs. These findings may be useful for intercollegiate special interest groups who are driving the development of integrated perioperative medicine [23, 24].

Similar to previous survey studies the most commonly identified barrier to developing perioperative medicine services was lack of funding for staffing [14, 15]. Our in-depth interviews also highlighted contextual barriers including geriatric medicine encroachment on other specialties in particular general medicine, and the competing priority of providing other geriatrics services such as community services. A notable barrier at the health professional level was the geriatricians’ concern that there was a lack of clarity about each specialty’s role, which could lead to duplicating or undervaluing a geriatrician’s unique skills. While team innovations can improve communication and satisfaction, poor understanding of roles can lead to conflict and confusion [25], and impact on patient safety [26]. It is therefore important as new team-based models of perioperative care are developed, clear role definitions are established to facilitate successful interprofessional collaborations [27, 28]. To inform the development of these multispecialty collaborations, further qualitative research is needed to explore in-depth the views and experiences of surgeons, anaesthetists and internal physicians. Further, fundamental to all healthcare services is patient-centredness. Application of patient-reported measures and qualitative research can be used to understand and engage patients in the development of perioperative services [29, 30].

Meeting the distinct and complex needs of older surgical patients emerged as a key facilitator. Geriatricians felt current perioperative medicine services did not provide continuity of care, and geriatric syndromes were not managed well. At a healthcare professional level, geriatricians perceived their skill set was unique and valuable for the care of older surgical patients. A recent study showed that implementation of a CGA toolkit to help non-geriatricians deliver CGA for older surgical patients was poorly taken up and implemented, despite buy in from surgeons and anaesthetists at the study hospitals [31]. This echoes the geriatricians’ view that they have a specific unique skill set that complements the skills of surgeons and anaesthetists. We also found that geriatricians wanted to support clinical geriatrics exposure for interested anaesthetists. There is an important need for training the general workforce in the principles of geriatric medicine, especially to reduce iatrogenic harm in older hospitalised adults, however, this does not replace the role of geriatricians [32, 33].

Despite the stated barriers, several geriatricians are already working with other specialties to develop collaborative perioperative medicine services. There were mixed opinions on whether to start with an inpatient or preadmission outpatient service. There is growing evidence for both inpatient [34–37] and outpatient geriatrics perioperative models of care [16]. However, translating clinical trial models of care into the real world healthcare setting is challenging and limited by resource availability. Staffing to deliver blanket consultation services is not available at most hospitals. This study identified alternative proactive integrated models of care that may be implemented with less staffing. For example, models based on proactive case-finding or screening-based referrals. A recent study found that a screening-based targeted frailty pathway in older surgical patients reduced readmissions and functional decline [38]. Due to the inherent subjective nature of some frailty tools, their interrater reliability needs to be established for use by non-geriatricians [39]. Geriatricians called for more data to inform how clinical trial models could be implemented in smaller hospitals with less resources. Implementation studies, local audits and quality improvement projects may help address this research void.

While our study was undertaken across two countries, our findings may not be generalisable to other countries. However, given the development of perioperative medicine is a global movement [6], the findings are likely to resonate with geriatricians in several countries. Like other qualitative studies, the findings may not be representative of the whole geriatrician population, and this study predominantly included heads of geriatric medicine department, although we actively sought a wide range of perspectives. The interviewer (JT) was a geriatrician and this could potentially have introduced bias, but this was minimised through the use of a semi-structured interview, a pre-determined interview guide, and inclusion of an expert in qualitative methodology (JJ) who was not a geriatrician. The consistency of responses also makes this bias less likely.

## Conclusions

Given national and international recommendations for collaborative models of perioperative care, there is a need to understand the current challenges and opportunities for delivering this care. This study demonstrated that geriatricians perceive several system and professional barriers to developing and delivering perioperative care services. Of equal importance to structural and process related challenges are the behaviour and attitudinal aspects that can either facilitate or block progress. There is a need to clarify the roles of each specialty to ensure a cohesive team. Geriatricians emphasised the importance of their unique skills for perioperative care. Despite a number of complex barriers, geriatricians are contemplating and implementing a variety of collaborative models of perioperative care. There is a need for strong infrastructure including geriatric medicine training opportunities for other disciplines, and a pragmatic evidence base to guide service development and delivery. Further research to understand the perspectives of other perioperative clinicians and patients, may help address barriers and progress perioperative service development.

## Supplementary Information

**Additional file 1.**

## Data Availability

The data that support the findings of this study are available on request from the corresponding author [JT]. The data are not publicly available due to them containing information that could compromise research participant privacy.

## Abbreviations

CGA: comprehensive geriatric assessment and management
POPS: proactive care of older patients undergoing surgery service
