# Supplementary material for: Geriatrician perspectives on perioperative care: a qualitative study
Source: BMC Geriatr. 2021 Jan 19;21:68. doi: 10.1186/s12877-021-02019-x (PMC7816344; doi:10.1186/s12877-021-02019-x)
Supplement: Supplementary file 1 — Additional file 1. [file 12877_2021_2019_MOESM1_ESM.pdf]

Supplementary File 1 - Interview guide

Main questions

1. *Do you have any questions before we begin?*
2. *To start off with, can you tell me how many years have you been working as a consultant geriatrician?*
3. *Do you consult or care for older surgical patients as part of your current duties?*
4. *Can you tell me more about the services that your Geriatric Medicine department currently provide for older surgical patients?*
5. *In the survey, you indicated that you provide services for surgical patients other than orthopaedic patients. Can you tell me more about this service? OPTIONAL*
   Other specific prompt questions:
   - *How long has this service been running for?*
   - *What works best about this service?*
   - *With more resources what would you do differently as part of this service?*
   - *Who currently funds this service?*
   - *What does this funding pay more?*
   - *Are there plans to extend this service to other surgical specialities?*
   - *Do you think other surgical specialities need this type of service?*
6. *Are you aware of other departments at your hospital that provide a surgical-medicine liaison service (including for orthopaedics), for example, general medicine? To which surgical specialities do these departments provide a surgical-medicine liaison service for?*
7. *In our survey we have described these type of services that extend beyond the standard consult model, as "surgical-geriatric medicine liaison services". Other terms in the literature include "Proactive care of older people undergoing surgery". What do you call these type of services?*
8. *In terms of other perioperative pathways, have you heard of ERAS (Enhanced Recovery After Surgery)? What do you know about ERAS?*
   Other specific prompt questions if ERAS program is running at their hospital:
   - *Who runs ERAS? Which patients is it provided for?*
   - *How well does ERAS meet the care needs of older surgical patients?*
   - *Should geriatricians be running ERAS or be developing their own service?*
9. *Perioperative medicine is a growing area. Do you think all geriatricians need training in perioperative medicine?*
   - *What do you think are some ways forward?*
   - *Should perioperative medicine be a formal post graduate qualification? Who could be eligible? i.e. geriatricians, intensivist, surgeons, anaesthetists, other physicians, GPs*
10. *In the survey, you indicated that geriatric medicine as a speciality needs to provide greater input into the care of older surgical patients. What do you think need to be our first steps towards this?*
    Other specific prompt questions:
    - *What do you see as the priority areas?*
    - *What are some of the current barriers?*
    - *Would you start with an inpatient or outpatient service if you had the resources? Why?*
    - *Which surgical specialty do you think (or why do you think the surgical specialty you picked on the survey) has the greatest need for geriatric medicine input?*

OR In the survey, you indicated that geriatric medicine as a speciality does not need to provide greater input into the care of older surgical patients. Do you think there are other priority areas? Can you tell me what you think these priority areas are?

Conclusion

1. *That brings us to the end of the interview. Thank you very much for participating. Did you want to say anything else or ask any questions?*
2. *Thank you again. Goodbye.*
